# Does a 2-Week Sexual Health in Rehabilitation Course Lead to Sustained Change in Students’ Attitudes?—A Pilot Study

**DOI:** 10.1007/s11195-018-9540-1

**Published:** 2018-10-22

**Authors:** H. Gerbild, C. M. Larsen, B. Rolander, Kristina Areskoug-Josefsson

**Affiliations:** 1 Research Unit of General Practice, Institute of Public Health, University of Southern Denmark, Odense, Denmark; 2 Department of Physiotherapy, University College Lillebaelt, Odense, Denmark; 3 Health Sciences Research Center, University College Lillebaelt, Odense, Denmark; 4 Department of Sports Science and Clinical Biomechanics, University of Southern Denmark, Odense, Denmark; 5 Futurum, Academy for Health and Care, Jönköping County Council, Jönköping, Sweden; 6 Department of Behavioural Science and Social Work, School of Health Sciences, Jönköping University, Jönköping, Sweden; 7 School of Health and Welfare, The Jönköping Academy for Improvement of Health and Welfare, Jönköping University, Jönköping, Sweden; 8 Department of Behavioural Sciences, Faculty of Health Sciences, Oslo Metropolitan University, Oslo, Norway

**Keywords:** Health professional students, Attitudes, Sexual health, Education, Denmark

## Abstract

This pilot study aimed to explore if healthcare professional students participating in a 2-week elective course, Sexual Health in Rehabilitation (SHR), led to significant and sustained change in experienced competence and attitudes towards addressing sexual health in their future professions, when measured with the Students’ Attitudes towards Sexual Health-Danish version (SA-SH-D). Comparison-group design, using the SA-SH-D at baseline, after the 2 weeks course and 3 months after completing the course. Participation in the SHR course significantly changed the students’ attitudes; decreasing their fears of offending the patients and increasing their feelings of comfort in communicating about sexual health, and the results sustained during the follow-up period of 3 months. The results of the intervention suggest that a 2-week elective SHR course leads to sustained change healthcare students’ attitudes towards addressing sexual health in their future profession. Sexual health education positively changed the students’ attitudes, decreased their fears of offending the patients and increased their feelings of comfort in communicating about sexual health. The SA-SH-D is a useful tool to measure results of educational interventions aiming to change healthcare students’ attitudes towards addressing sexual health in their future profession. Future research is recommended regarding students’ attitudes towards addressing sexual health with persons living with disabilities. There is also a need to further research the effect of elective versus compulsory sexual health education in healthcare programs, to lessen the risk that healthcare students in their future profession will not be able to give equal care due to variation in competence and attitude.

## Introduction

Sexual health is an important part of general health, implying the importance of promoting sexual health as a healthcare professional [1–3]. Healthcare professionals should be competent, confident and supportive when addressing sexual health, even if patients’ expectations of healthcare professionals’ competence regarding sexual health often are low [4–6]. Professional’s attitudes, confidence and beliefs regarding sexual health also affect how healthcare professionals work towards improving sexual health [7–9]. Previous research has shown a widespread lack of education concerning sexual health and personal attitudes together with the belief that sexual health is not important for persons with disease or disability, which may further problematize promotion of sexual health among healthcare professionals [10, 11]. Healthcare professionals and healthcare professional students agree that sexual health could be part of care and rehabilitation for patients, but that they experience lack of education concerning sexual health and communication about sexual health issues [12–16]. Healthcare students often have a high level of discomfort concerning communicating about sexual issues, implying the importance of sexual health education and communication about sensitive topics [17, 18].

To ensure that the educational interventions in sexual health suits the healthcare professional students’ educational needs, the design of the educational program should take into account available evidence regarding didactics in sexual health education, since the design and pedagogical model will affect the outcome of the intervention [13, 16, 19–21]. In some instances, sexuality education programs are offered as electives in the health professional curriculum [22] and to ensure the outcome of the sexual health education intervention, it is essential to measure the results of the educational intervention, as well as if there are sustainable differences in the student’s attitudes towards addressing sexual health in their future profession depending participation in an elective sexuality education course. To our knowledge there have been no studies evaluating if healthcare professional students who have chosen to participate in sexual health courses and those who have chosen other elective courses, have significant differences in experienced competence and attitudes towards addressing sexual health in their future professions. The Students’ Attitudes towards Sexual Health is a valid and reliable questionnaire [23, 24], but it has not yet been used to evaluate educational interventions. This pilot study was performed in Denmark, where there are several national rehabilitation guidelines for various diseases and functional disabilities that include sexual health. Those guidelines denote that Danish healthcare professionals must be prepared to address sexual health [24].

## Aim

The aim of this pilot study was to explore if healthcare professional students participating in an elective sexual health education course led to significant and sustained change in experienced competence and attitudes towards addressing sexual health in their future professions, when measured with the Students’ Attitudes towards Sexual Health-Danish version (SA-SH-D).

## Methods

### Study Design

A comparison-group study design was used. The outcome of a sexual health educational intervention was measured with the Students’ Attitudes towards Sexual Health (Danish Version) (SA-SH-D) questionnaire [24], distributed at baseline, after the 2-week elective course and at 3-months’ follow-up after the course.

### Participants

The participating students were in the final year of their basic education in a health science program in Denmark, studying to become nurses, radiographic nurses, physiotherapists or occupational therapists, where an elective 2-week course is included in their educational programs. The participating students in the intervention group had chosen the elective course of sexual health in rehabilitation (SHR), and in the comparison-group, the students had chosen the elective course of project leadership (PL). The elective courses where performed during the same time period. The students were informed of the research project after they had made their choices regarding the elective courses. All students from both courses were invited to participate in the study. At baseline 23 of 25 students in the SHR-course participated (92% response rate), as did 17 of 19 students in the PL-course (89% response rate). The sample size in this pilot study was defined by the size of the educational group, and the number of students in each course depended on the students’ own choice of elective course.

### Questionnaire

The SA-SH-D consists of 22 items to be answered on a Likert scale with five options: disagree, partly disagree, partly agree, agree and strongly agree. The responses ‘totally agree/partly agree’ are considered positive for positively loaded items, and for negatively loaded items the responses ‘disagree/partly disagree’ are considered as showing a positive attitude). The psychometric testing of the SA-SH-D showed a Cronbach’s alpha of 0.67, high relevance for content validity index (item context validity index 0.82–1.0) and satisfactory item scale correlation [24]. The factors included in the factor analysis of original Swedish version of the SA-SH were *present feelings of comfortableness* (questions 1–8), *future working environment* (questions 14, 17, 18), and *fear of negative influence on future patient relations* (questions 9, 10, 13) [12]. In both the SA-SH and the SA-SH-D, questions 11, 12, 15, 16 and 19–22 are not included in any of the factors, but are still retained in the questionnaire, since they were considered relevant [12, 24]. Descriptive questions regarding gender, age, educational program and educational level within the program, were added to the questionnaire.

### Procedure

The participants answered the SA-SH-D three times: the first day of the course (baseline), the last day of the 2-week course (follow-up 1) and 3 months after finishing the course (follow-up 2). The questionnaire was paper-based and handed out in the classroom at the baseline and follow-up 1. Follow-up 2 was performed by e-mail, since the students had finished their educational program at the time of the third data collection. The data collection procedure was identical in both participating groups. The teachers of the courses were not informed of the results of the baseline data or if the students participated in the study or not.

### Elective Course: Sexual Health in Rehabilitation

The course was a 2-week full-time university course about sexual health based on research on sexual health and rehabilitation. The course was inter-professional and attentive to the students’ own attitudes towards sexual health, which has been recommended for sexual health educational interventions [16, 19, 25, 26].

The SHR course consisted of the following concepts:

- sexuality—definition, concept and rights
- sexual anatomy and physiology
- sexual functioning and the sexual response cycle
- sex-related problems caused by disease, trauma and treatment
- consequences and late effects of diseases of importance to sexuality
- sexuality in different life stages
- cultural and social barriers
- body awareness, sensuality and intimacy
- norms, values and attitudes to sexuality
- sexual aids
- sexology counselling and intervention
- professional responsibilities and areas of intervention

The first part of the course focused on the first three concepts to give the students basic knowledge of sexual health. The second part concerned sexual health problems and the third part focused on promoting sexual health. The course had 35 educational sessions (45 min/session) including various pedagogical methods, such as lectures, seminars, inter-professional study groups, supervision, oral and written student presentations, peer and teacher feedback and reading assignments.

### Data Analysis

Descriptive statistics were presented as means, medians, percentiles (25, 75) and cumulative percentages.

A nonparametric analysis for repeated measures, Friedman’s test (method = exact), was used to compare the baseline data with the two sets of follow-up data measured after 2 weeks and 3 months, respectively. When a significant difference according to Friedman’s test (method = exact) was found, additional tests with Wilcoxon (method = exact) were performed to identify specific differences.

The results of the groups (SHR and PL) were compared with Mann–Whitney U test (method = exact). The exact method was used for all analyses due to the small sample size. The limit of statistical significance was set at α = 0.01. The statistical analyses were performed with IBM SPSS version 22 (IBM Corp., Armonk, NY, USA).

## Ethical Approval

Ethical issues were considered, and the project received ethical approval by the Danish Data Protection Agency. All procedures performed in the study were in accordance with the ethical standards of the institutional and/or national research committee and with the 1964 Helsinki declaration and its later amendments or comparable ethical standards. All participants gave their informed consent to participate in the research project by marking in the affirmative on a specific question regarding informed consent on the questionnaire. All the collected data were anonymous to the analysing researchers.

## Results

**Fig. 1:**
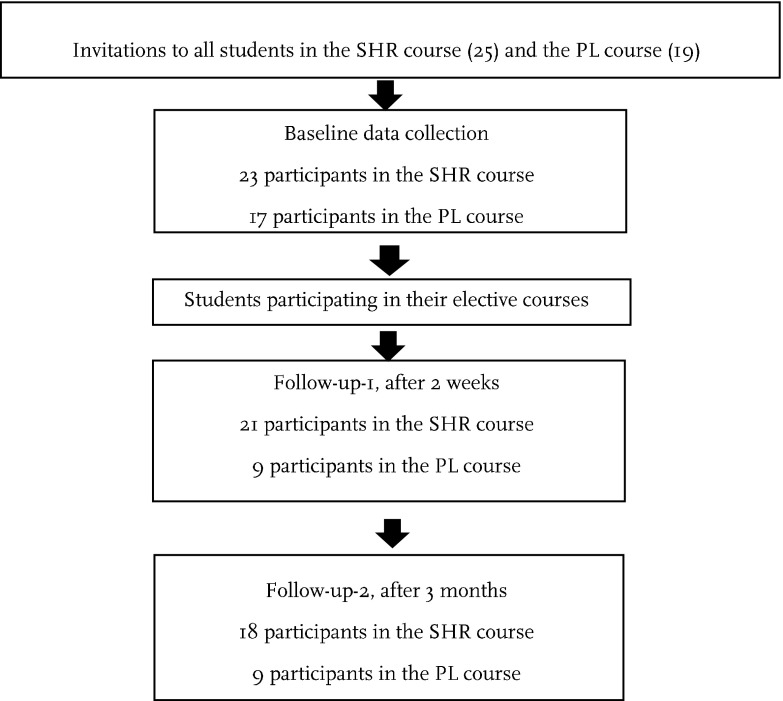
Flowchart of data collection process

There was a decrease in participation at the both follow-ups (Fig. 1). The participating groups (SHR and PL) were similar regarding age and gender but varied by future profession (Table 1).

**Table 1:** Descriptive statistics of participants in the SHR and PL groups, as number (n), percentage (%), mean and standard deviation (SD)

	Baseline		Follow-up 1		Follow-up 2	
	SHR	PL	SHR	PL	SHR	PL
	n	n	n	n	n	n
Gender						
Men	1	1	1	1	1	1
Women	22	16	20	8	17	8
Missing	–		2 (9%)	8 (47%)	4 (18%)	8 (47%)
Educational programme						
Physiotherapy	7	1	7	1	6	1
Occupational therapy	15	6	13	3	11	3
Nursing	1	9	1	4	1	4
Missing	–	1 (6%)	2 (9%)	8 (47%)	5 (22%)	8 (47%)
Age (mean ± SD)	25 ± 3	27 ± 6	25 ± 3	27 ± 7	25 ± 3	25 ± 3

All participants in the sexual health course completed the course.

The students choosing to participate in the SHR course were not more prepared to address sexual health than students in the PL group. The few significant differences at baseline between the SHR and the PL group were found for the following questions:

Question 4 *I feel comfortable about discussing sexual health issues with future patients regardless of their sex*, where the SHR group had a more positive attitude than the PL group (*p* = 0.045).

Question 7 *I feel comfortable about discussing sexual health issues with future patients regardless of their sexual orientation*, where the PL group had a more positive attitude than the SHR group (*p* = 0.007).

Question 20 *I think that I as a student need to get basic knowledge about sexual health in my education*, where the SHR group had a more positive attitude than the PL group (*p* = 0.01).

Regardless of group, all students showed low perceived ability in communicating about sexual health with patients and low perceived competence and educational level in the field, prior to the intervention (Tables 2, 3, 4, 5). The baseline measurement also shows the students’ awareness of their need to be further educated regarding sexual health and communication about sexual health. The students also expressed worry about embarrassing the patients and fear of creating a negative patient–health professional relation if they addressed sexual health issues.

**Table 2:** Medians, percentiles (25, 75) and *p* values for each question in factor 1 at baseline, follow-up 1 and follow-up 2

Question	Baseline			Follow-up 1			Follow-up 2		
	SHR median, (percentiles 25, 75)	PL median, (percentiles 25, 75)	Differences between groups, *p* value	SHR median, (percentiles 25, 75)	PL median, (percentiles 25, 75)	Differences between groups, *p* value	SHR median, (percentiles 25, 75)	PL median, (percentiles 25, 75)	Differences between groups, *p* value
1. I feel comfortable about informing future patients about sexual health	3.0^1,2^ (2.0–3.0)	3.0 (2.5–4.0)	0.16	4.0^1***^ (3.0–4.0)	3.0 (1.5–4.0)	0.06	4.0^2***^ (3.0–4.0)	2.0 (1.0–3.5)	0.01
2. I feel comfortable about initiating a conversation regarding sexual health with future patients	2.0^3,4^ (2.0–3.0)	3.0 (2.0–3.0)	0.16	4.0^3***^ (3.5–4.0)	3.0 (1.5–3.0)	0.001	4.0^4***^ (3.0–4.0)	2.0 (1.5–3.0)	0.001
3. I feel comfortable about discussing sexual health with future patients	3.0^5,6^ (2.0–3.0)	3.0 (3.0–3.0)	0.18	4.0^5***^ (3.0–4.0)	3.0 (2.0–4.0)	0.07	4.0^6**^ (3.0–4.0)	3.0 (1.5–3.5)	0.02
4. I feel comfortable about discussing sexual health issues with future patients regardless of their sex	2.0^7,8^ (2.0–3.0)	3.0 (3.0–3.5)	0.045	4.0^7***^ (3.0–4.0)	3.0 (1.5–3.5)	0.02	4.0^8***^ (3.0–4.0)	3.0 (1.5–3.5)	0.01
5. I feel comfortable about discussing sexual health issues with future patients regardless of their age	2.0^9,10^ (2.0–3.0)	3.0 (2.5–3.5)	0.11	4.0^9***^ (3.0–4.0)	3.0 (1.5–4.0)	0.03	4.0^10**^ (3.0–4.0)	3.0 (2.0–3.5)	0.02
6. I feel comfortable about discussing sexual health issues with future patients regardless of their cultural background	2.0^11,12^ (1.0–3.0)	2.0 (2.0–2.5)	0.94	3.0^11***^ (3.0–4.0)	2.0 (1.0–3.0)	0.01	3.0^12***^ (3.0–4.0)	1.0 (1.0–2.0)	< 0.001
7. I feel comfortable about discussing sexual health issues with future patients regardless of their sexual orientation	3.0^13,14^ (2.0–3.0)	3.0 (3.0–4.0)	0.01	4.0^13***^ (3.0–4.0)	3.0 (2.0–4.0)	0.14	3.0^14**^ (3.0–4.0)	3.0 (2.0–4.0)	0.11
8. I feel comfortable about discussing specific sexual activities with future patients	2.0^15,16^ (1.0–2.0)	2.0 (2.0–3.0)	0.14	4.0^15***^ (3.0–4.0)	2.0 (1.5–3.5)	0.01	3.0^16**^ (3.0–4.0)	2.0 (1.0–2.5)	0.001

**p < 0.01, ***p < 0.001 shows significant differences between baseline, follow-up after 2 weeks and follow-up after 3 months

**Table 3:** Medians, percentiles (25, 75) and *p* values for each question in factor 2 at baseline, follow-up 1 and follow-up 2

Question	Baseline			Follow-up 1			Follow-up 2		
	SHR median, (percentiles 25, 75)	PL median, (percentiles 25, 75)	Differences between groups, *p* value	SHR median, (percentiles 25, 75)	PL median, (percentiles 25, 75)	Differences between groups, *p* value	SHR median, (percentiles 25, 75)	PL median, (percentiles 25, 75)	Differences between groups, *p* value
14. I believe that I will have too much to do in my future profession to have time to handle sexual issues.	4.0 (3.0–5.0)	3.0 (3.0–4.0)	0.10	4.0 (3.0–4.0)	3.0 (2.5–4.0)	0.13	4.0 (3.0–4.0)	3.0 (2.5–4.0)	0.29
17. I am afraid that my future colleagues would feel uncomfortable in dealing with questions regarding patients’ sexual health	3.0 (3.0–4.0)	3.0 (2.0–4.0)	0.75	3.0 (3.0–4.0)	3.0 (3.0–4.0)	0.69	3.0 (3.0–4.0)	3.0 (2.0–3.0)	0.36
18. I believe that my future colleagues will be reluctant to talk about sexual issues	3.0 (3.0–4.0)	3.0 (3.0–4.0)	0.42	3.0 (3.0–4.0)	4.0 (3.0–4.5)	0.50	3.0 (3.0–3.0)	3.0 (2.5–4.0)	0.67

**p < 0.01, ***p < 0.001 shows significant differences between baseline, follow-up after 2 weeks and follow-up after 3 months

**Table 4:** Medians, percentiles (25, 75) and p-values for each question in factor 3 at baseline, follow-up 1 and follow-up 2

Question	Baseline			Follow-up 1			Follow-up 2		
	SHR median, (percentiles 25, 75)	PL median, (percentiles 25, 75)	Differences between groups, *p* value	SHR median, (percentiles 25, 75)	PL median, (percentiles 25, 75)	Differences between groups, *p* value	SHR median, (percentiles 25, 75)	PL median, (percentiles 25, 75)	Differences between groups, *p* value
9. I am unprepared to talk about sexual health with future patients	2.5^1,2^ (2.0–3.0)	2.0 (1.5–3.5)	0.48	4.0^1**^ (3.5–4.0)	3.0 (2.0–4.0)	0.02	4.0^2**^ (3.0–4.0)	2.0 (1.5–2.5)	0.001
10. I believe that I might feel embarrassed if future patients talk about sexual issues	3.0^3,4^ (2.0–4.0)	3.0 (3.0–4.0)	0.42	4.0^3***^ (4.0–5.0)	3.0 (3.0–4.0)	0.03	4.0^4**^ (4.0–5.0)	3.0 (3.0–4.0)	0.01
13. I am afraid that conversa-tions regarding sexual health might create a distance between me and the patients	3.0^5,6^ (3.0–4.0)	3.0 (3.0–4.5)	0.53	4.0^5***^ (4.0–5.0)	4.0 (2.5–4.0)	0.02	4.0^6**^ (4.0–5.0)	2.0 (1.5–3.5)	0.001

**p < 0.01, ***p < 0.001 shows significant differences between baseline, follow-up after 2 weeks and follow-up after 3 months

**Table 5:** Medians, percentiles (25, 75) and *p* values for each question that was not included in the factors, at baseline, follow-up 1 and follow-up 2

Question	Baseline			Follow-up 1			Follow-up 2		
	SHR median, (percentiles 25, 75)	PL median, (percentiles 25, 75)	Differences between groups, *p* value	SHR median, (percentiles 25, 75)	PL median, (percentiles 25, 75)	Differences between groups, *p* value	SHR median, (percentiles 25, 75)	PL median, (percentiles 25, 75)	Differences between groups, *p* value
11. I believe that future patients might feel embarrassed if I bring up sexual issues	3.0^1***^ (2.0–3.0)	2.0 (2.0–3.0)	0.40	4.0^1,2^ (3.0–4.0)	3.0 (1.5–3.0)	0.004	3.0^2**^ (3.0–3.0)	2.0 (1.5–3.0)	0.08
12. I am afraid that future patients might feel uneasy if I talk about sexual issues	3.0^3,4^ (2.0–3.0)	3.0 (2.0–4.0)	0.81	4.0^3***^ (3.5–4.0)	3.0 (1.5–3.5)	0.005	4.0^4**^ (3.0–4.0)	2.0 (1.5–3.0)	0.002
15. I will take time to deal with patients’ sexual issues in my future profession	3.0 (2.0–4.0)	3.0 (3.0–4.0)	0.11	4.0 (4.0–4.0)	3.0 (2.0–4.0)	0.03	4.0 (3.0–5.0)	3.0 (2.5–3.5)	0.01
16. I am afraid that my future colleagues would feel uneasy if I brought up sexual issues with patients	4.0 (3.0–5.0)	4.0 (3.0–5.0)	0.53	4.0 (3.0–5.0)	4.0 (3.5–5.0)	0.73	4.0 (3.0–5.0)	3.0 (2.5–4.0)	0.07
19. In my education I have been educated about sexual health	2.0^5,6^ (1.0–2.0)	2.0 (1.0–3.0)	0.32	4.0^5***,7^ (3.0–4.0)	2.0 (1.0–2.0)	<0.001	3.0^6**,7*^ (3.0–4.0)	2.0 (1.0–2.0)	< 0.001
20. I think that I as a student need to get basic knowledge about sexual health in my education	5.0 (4.0–5.0)	4.0 (3.0–5.0)	0.01	5.0 (4.0–5.0)	4.0 (4.0–5.0)	0.30	5.0 (5.0–5.0)	5.0 (3.0–5.0)	0.24
21. I have sufficient competence to talk about sexual health with my future patients	2.0^8,9^ (1.0–2.0)	2.0 (2.0–3.0)	0.13	3.0^8***^ (3.0–4.0)	2.0 (1.0–2.0)	<0.001	3.0^9***^ (3.0–4.0)	2.0 (1.5–2.0)	0.001
22. I think that I need to be trained in my education to talk about sexual health	5.0 (4.0–5.0)	4.0 (4.0–5.0)	0.10	5.0 (5.0–5.0)	4.0 (4.0–5.0)	0.05	5.0 (5.0–5.0)	5.0 (3.5–5.0)	0.24

*p < 0.05, **p < 0.01, ***p < 0.001 shows significant differences between baseline, follow-up after 2 weeks and follow-up after 3 months

All questions in factor 1 (*present feelings of comfortableness*) show that participating in the SHR course had a positive influence on feelings of comfort in addressing sexual health with patients (Table 2).

None of the questions in factor 2 (*future working environment*) showed effect of attending the SHR course, which was expected, since those issues weren’t thought to be affected by the intervention (Table 3).

All questions in factor 3 (*fear of negative influence on future patient relations*), showed positive effects of participating in the SHR course (Table 4).

Among the questions not included in the factors 1–3, question 11 (I believe that future patients might feel embarrassed if I bring up sexual issues), had a change over time in both groups, suggesting instability of the question (Table 5). Questions 12, 19 and 21 showed a positive effect in the SHR group. Questions 15, 16, 20 and 22 showed no significant differences after participating in the SHR course (Table 5).

By looking at the level of positive attitudes towards working with sexual health in their future profession at baseline and at follow-ups 1 and 2, it is evident that there might be a fluctuation over time, but in this pilot study the outcome of participating in the SHR course is clear compared to participating in another elective course in the educational program and the results sustain 3 months after participation in the course (Tables 2, 3, 4 and 5). The largest change in positive attitudes is in factor 1 (*present feelings of comfortableness*, Q1–Q8) (Table 2), which was rated very low by both groups at baseline.

## Discussion

The few differences between the groups at baseline indicate that making an active choice to attend a SHR course does not mean that those students were more prepared to address sexual health with patients. Since the topic of sexual health is quite novel in rehabilitation it is of interest to note that there were more students from occupational therapy and physiotherapy, than nursing students, choosing the course in sexual health. The reasons could be interesting to research further, since they may vary from students having a specific interest in the field or knowledge of lack of competence in the field, to more pragmatic reasons, such as the popularity of teachers.

There was a tendency for improvement in both groups after the 2-week courses. This may be due to the students having had informal meetings where educational issues and topics may have been discussed, thereby causing a reflection regarding sexology issues also for the students in the PL group. However, the fact that the students in the comparison group (PL) did not get education or encouragement to reflect further on those issues could explain why this positive side effect didn’t persist in the 3-month follow-up.

The largest changes after the educational intervention were for the questions in factor 1, present feelings of comfortableness (together with question 21: *I have sufficient competence to talk about sexual health with my future patients*), indicating that the elective SHR course increased feelings of comfort regarding addressing sexual health with patients. Patients prefer to communicate about sexual health with healthcare professionals who are competent and comfortable in discussing sexual issues, which implies the importance of those results [6]. Persons with a more inclusive sexual orientation often experience negative communication with healthcare professionals [27], and an improvement in this field addressing sexual health is therefore probably beneficial, if the student has gained and experience competence in addressing persons with a more inclusive sexual orientation in a better way. There are recommendations that healthcare professional students should be educated in more inclusive sexual orientation issues early in their education [27]; thus, it is possible that this study would result in even more positive results if the SHR course was performed earlier in the educational program. The positive effects of the SHR course on the questions in factor 3, *fear of negative influence on future patient relations* (together with question 12: *I am afraid that future patients might feel uneasy if I talk about sexual issues*), imply that the students had less fear of creating negative emotions in addressing sexual health in the clinical encounter. Those results are important, since embarrassment, fear of embarrassing the patient and risks of being seen as offensive by the patient, are known barriers to communicating about sexual health. The questions in factor 2, *future working environment* (together with question 16: *I am afraid that my future colleagues would feel uneasy if I brought up sexual issues with patients*) did not show significant change after the SHR course, which is reasonable, considering that the questions regard attitudes of future colleagues.

Since the results of this pilot study clearly show, that students who have gained additional education in sexual health issues will be better prepared, and therefore prone to give better healthcare regarding sexual health, than those students finishing their basic healthcare professional education without it. The students who have not been educated in addressing sexual health and sexual issues may be more likely to neglect this field in clinical encounters due to perceived negative barriers and lack of competence [9]. The neglect of sexual health in healthcare and in healthcare professionals’ education has been shown in earlier research to have negative effect on the health of patients [9]. Thus, the results of this pilot study imply the importance of including sexual health and sexology as a compulsory part of the curricula for healthcare professionals’ educational programs, instead of as elective courses. A compulsory inclusion of sexology and sexual health in the curricula would also make a statement to the students about the importance of sexual health promotion as being part of their future profession. Legitimizing of sexual health in healthcare professionals’ education may be especially important in professions where sexual health is a novel area, such as for example physiotherapy and occupational therapy [7, 28, 29], sending a clear message that healthcare professionals are expected to promote sexual health, and that this competence is part of the expected professional competence.

The educational intervention included themes connected to living with various disabilities through the lifespan and it is probable that the intervention may have decreased potential barriers towards addressing sexual health for persons living with disabilities. However, there is a need to further research students’ attitudes towards sexual health for persons living with disabilities, considering that the SA-SH does not include this theme.

The SHR course was only 2 weeks, and it is interesting that the outcome of the course was sustained at the 3-month follow-up. In previous studies of educational interventions in sexual health, there has been an effect of the educational interventions, but the educational interventions have been of longer duration, and the follow-up time has only been a few weeks after finalizing the course [25]. In future research, it would be of interest to follow the participants over a longer time span, to explore whether the positive effects of SHR courses also show long-lasting change.

In this pilot study, it was evident that a 2-week elective SHR course can assist students in a change of attitudes towards working with and addressing sexual health in their future professions and also that the effect of the educational intervention lasts for at least 3 months after the end of the course. Those results are interesting, considering that there are recommendations from previous research that sexual health educational interventions should be incorporated longitudinally throughout the curricula [13]. Further research is needed to establish the briefest educational intervention that can lead to sustainable changes in attitudes, competence and knowledge regarding sexual health among students in healthcare professions.

In this elective course, the pedagogical intent was to activate the students, and to aid the students to reflect more deeply on sexual health issues and then to create useful knowledge and practical skills in sexual health in rehabilitation. Other pedagogical models may lead to other results, even if the education consisted of similar content. There are recommendations that sexual health education should be inter-professional and include training in practical communication skills to gain best results, and the positive results of this pilot study seem to support this recommendation [6, 9, 20].

This study did not discuss the potential differences in attitudes towards promoting sexual health according to future profession, which have been shown in previous research [30]. The effect of future profession was not explored due to the small sample size for each profession. The small sample size and the drop-out rate at follow-up, could also have affected the results of this pilot study, but since all students in the available group were invited, and the baseline response rate was high, the results are considered to be of value.

In the psychometric study of the SA-SH-D ceiling effects were shown for questions 20 and 22. The ceiling effects are possibly the reason why those two questions did not show significantly better results after participating in the SHR course. It is interesting that both groups rated questions 20 and 22 high, indicating that the students found it very important to get increased education regarding sexual health and regarding communication about sexual health with patients. This implies that both groups would have liked to have the SHR course in their basic education. The results are in line with earlier results regarding experiences of educational needs in sexology and sexual health [7, 17, 30].

There seemed to be instability regarding question 11 (*I believe that future patients might feel embarrassed if I bring up sexual issues*), and further research in larger sample sizes is needed to uncover potential reasons for the instability of the answers.

A majority of the participants being women in this sample is representative to the population of students in healthcare professions in Denmark, but there may be gender differences regarding attitudes towards working with sexual health, which should be considered in future studies.

Although the sample size was small in this study, the respondents are a large majority of the potential participants, indicating that the sample can be representative. In addition, due to the number of analyses conducted to respond to the aim of the study, the risk of erroneous significant relationships (Type 1 error) has been addressed. With *p* = 0.05, the calculated risk is much more than 100% for Type 1 errors according to Bonferroni and Hochberg. Consequently, the overall alpha level should be lowered to compensate for this risk. But corrections made in accordance with Bonferroni and Hochberg will often be too conservative, in this case for all analyses between *p* = 0.002 and *p* = 0.0007, and will increase the statistical Type II error, that is, retaining the null hypothesis when it should have been rejected [31–33]. Therefore, the significance level chosen was α = 0.01.

## Conclusion

The results of the intervention suggest that a 2-week elective SHR course leads to sustained change in healthcare students’ attitudes towards addressing sexual health in their future profession. Sexual health education positively changed the students’ attitudes, decreased their fears of offending the patients and increased their feelings of comfort in communicating about sexual health. The results of the educational intervention were sustained over 3 months.

The SA-SH-D is a useful tool to measure results of educational interventions aiming to change healthcare students’ attitudes towards addressing sexual health in their future profession. Future research is recommended regarding students’ attitudes towards addressing sexual health with persons living with disabilities.

There is also a need to further research the effect of elective versus compulsory sexual health education in healthcare programs, to lessen the risk that healthcare students in their future profession will not be able to give equal care due to variation in competence and attitude.
